# Collodion in Skin Diseases

**Published:** 1851-09

**Authors:** 


*Collodion in Skin Diseases.* — Collodion seems to act admirably in those cases where a kind of artificial epidermis is required. Dr. Spengler, in the *Neue Med. u. Chir. Zeitschrift*, No. 28, 1850, gives various cases where the collodion either led to the cure, or greatly relieved the symptoms. The author mentions that this substance was first used in skin diseases by Mr. Wilson, in this country; and gives details of the success he (the author) met with in thus treating impetigo, lichen, herpes labialis, chronic eczema, and cracked nipples. Our readers are aware that collodion is largely used in the London Hospitals in erysipelas of the face. — *London Lancet.*

